# A Conserved Role for Stomatin Domain Genes in Olfactory Behavior

**DOI:** 10.1523/ENEURO.0457-22.2023

**Published:** 2023-03-21

**Authors:** Xiaoyu Liang, Morgan Taylor, Rebekah Napier-Jameson, Canyon Calovich-Benne, Adam Norris

**Affiliations:** Department of Biological Sciences, Southern Methodist University, Dallas, Texas 75275

**Keywords:** behavior, bhemotaxis, msec-2, stomatin, stoml3, olfaction

## Abstract

The highly-conserved stomatin domain has been identified in genes throughout all classes of life. In animals, different stomatin domain-encoding genes have been implicated in the function of the kidney, red blood cells, and specific neuron types, although the underlying mechanisms remain unresolved. In one well-studied example of stomatin domain gene function, the *Caenorhabditis elegans* gene *mec-2* and its mouse homolog *Stoml3* are required for the function of mechanosensory neurons, where they modulate the activity of mechanosensory ion channels on the plasma membrane. Here, we identify an additional shared function for *mec-2* and *Stoml3* in a very different sensory context, that of olfaction. In worms, we find that a subset of stomatin domain genes are expressed in olfactory neurons, but only *mec-2* is strongly required for olfactory behavior. *mec-2* acts cell-autonomously and multiple alternatively-spliced isoforms of *mec-2* can be substituted for each other. We generate a *Stoml3* knock-out (KO) mouse and demonstrate that, like its worm homolog *mec-2*, it is required for olfactory behavior. In mice, *Stoml3* is not required for odor detection, but is required for odor discrimination. Therefore, in addition to their shared roles in mechanosensory behavior, *mec-2* and *Stoml3* also have a shared role in olfactory behavior.

## Significance Statement

In this manuscript we reveal a surprising moonlighting function for the conserved stomatin domain genes *mec-2* (worm) and *Stoml3* (mouse). Both factors have previously been studied extensively for their roles in mediating touch sensation in mechanosensory neurons. Here, we demonstrate that, in both worms and mice, they play an additional previously-unappreciated role in a different sensory modality, that of olfaction.

## Introduction

Olfaction is a remarkable sensory system, enabling animals to detect and distinguish among a very large range of odors, and at very low concentrations. A number of cellular and molecular aspects of the olfactory system of various animals have been well characterized, including odorant-receptor interactions, signal transduction cascades within olfactory neurons, and downstream neuronal circuits (Buck and Axel, 1991; Katada et al., 2005; Hart and Chao, 2010; Del Mármol et al., 2021). However, many factors that are highly expressed in olfactory neurons remain completely uncharacterized or with poorly defined function (Kobayakawa et al., 2002; Kanageswaran et al., 2015; Dibattista et al., 2021).

We recently found that the stomatin domain gene *mec-2* is highly expressed in *Caenorhabditis elegans* olfactory neurons, and is required for olfaction (Liang et al., 2022). The canonical role for *mec-2* and its mouse homolog *Stoml3* is as a component of the mechanotransduction machinery expressed in mechanosensory neurons (Huang et al., 1995; Wetzel et al., 2007). Our recent results therefore suggest that *mec-2*, and perhaps *Stoml3* or other stomatin domain genes, might have previously unappreciated roles in olfactory neurons.

**Figure 1.**
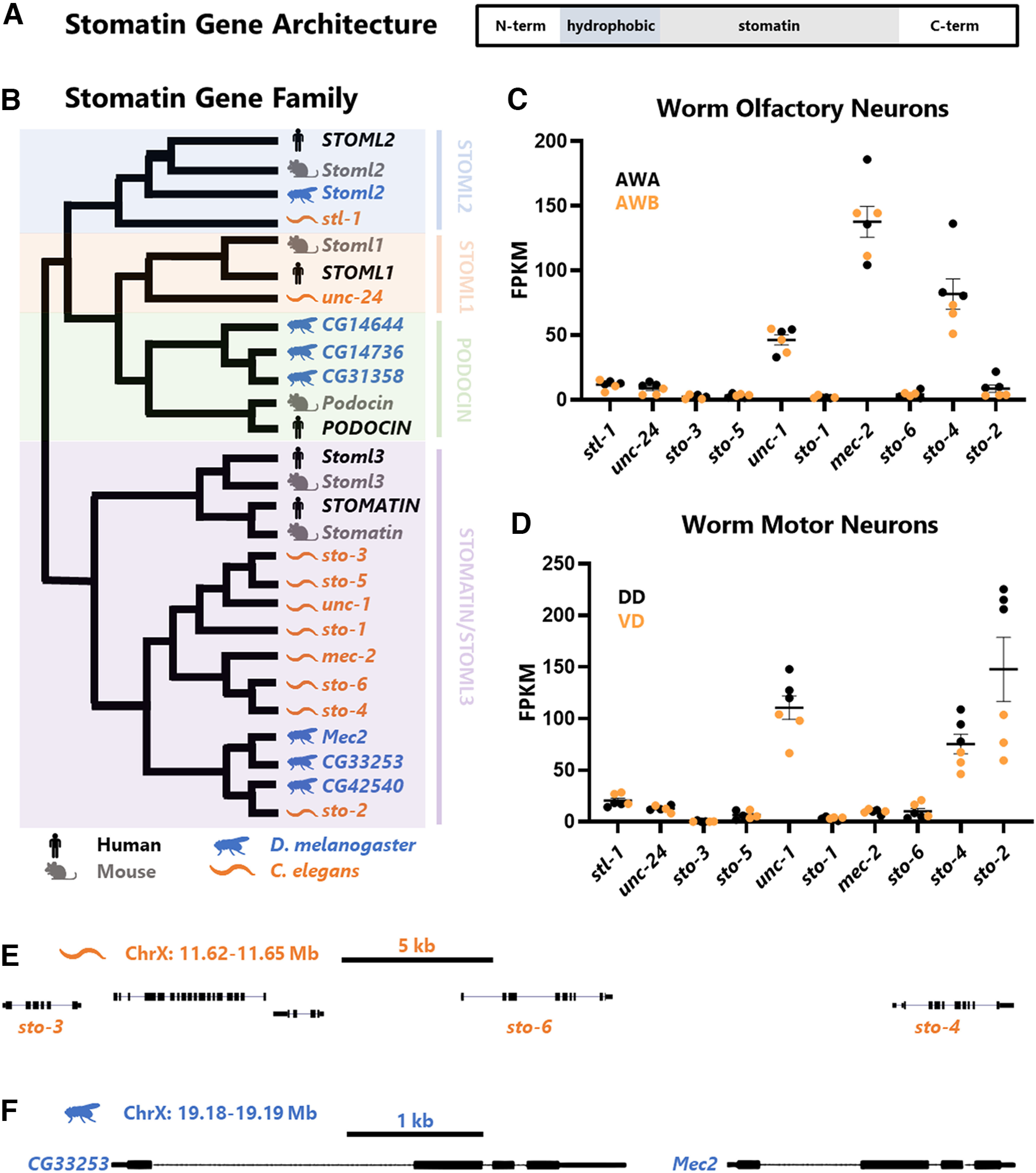
A subset of stomatin domain genes are expressed in *C. elegans* olfactory neurons. ***A***, Domain organization of stomatin domain genes. Colored in shades of gray are the central conserved stomatin domain and the hydrophobic domain that mediates association with the membrane. Flanking these are the divergent N and C termini. ***B***, Gene tree for family of stomatin domain gene homologues, TF105750 (stomatin-like 2) from TreeFam (Ruan et al., 2008). Some organism silhouettes created with BioRender. ***C***, ***D***, RNA Seq data from CenGEN consortium sorted neuron populations, mapped to the worm genome with STAR and gene reads counted by HTSeq. ***C***, Olfactory neurons AWA and AWB. ***D***, Motor neurons DD and VD. See also Extended Data Figure 1-1*A* for comparison with olfactory neuron marker gene FPKM. ***E***, Cluster of three *Stoml3/Stomatin*-like genes in the *C. elegans* genome on chromosome X. ***F***, Two immediately adjacent *Stoml3/Stomatin-*like genes in the *Drosophila* genome on chromosome X. See also Extended Data Figure 1-1.

*mec-2* and *Stoml3* belong to a family of proteins defined by the presence of a highly-conserved stomatin domain, which can be found across all domains of life (Green and Young, 2008). The typical architecture of stomatin domain genes entails a central conserved stomatin domain and a hydrophobic region that mediates membrane association (Fig. 1*A*). These are flanked by divergent N and C termini, which can confer specific functional and regulatory features (Rungaldier et al., 2017; Cullinan et al., 2021; Liang et al., 2022). Stomatin domain proteins localize to the plasma membrane via their hydrophobic region, where some family members have been shown to modulate the activity of membrane proteins, although the precise mechanisms by which they do so remain elusive (Goodman et al., 2002; Genetet et al., 2017; Cullinan et al., 2021).

10.1523/ENEURO.0457-22.2023.f1-1 Extended Data Figure 1-1 Expression and location of Stomatin domain gene homologues. ***A***, FPKMs for *osm-6* as a positive control for a gene highly expressed in olfactory neurons (it serves as a sensory neuron marker gene) and lowly expressed in other neurons, including motor neurons. ***B***, Additional cluster of *Drosophila Podocin* homologues on chromosome 3. Download Figure 1-1, TIF file.

**Figure 2.**
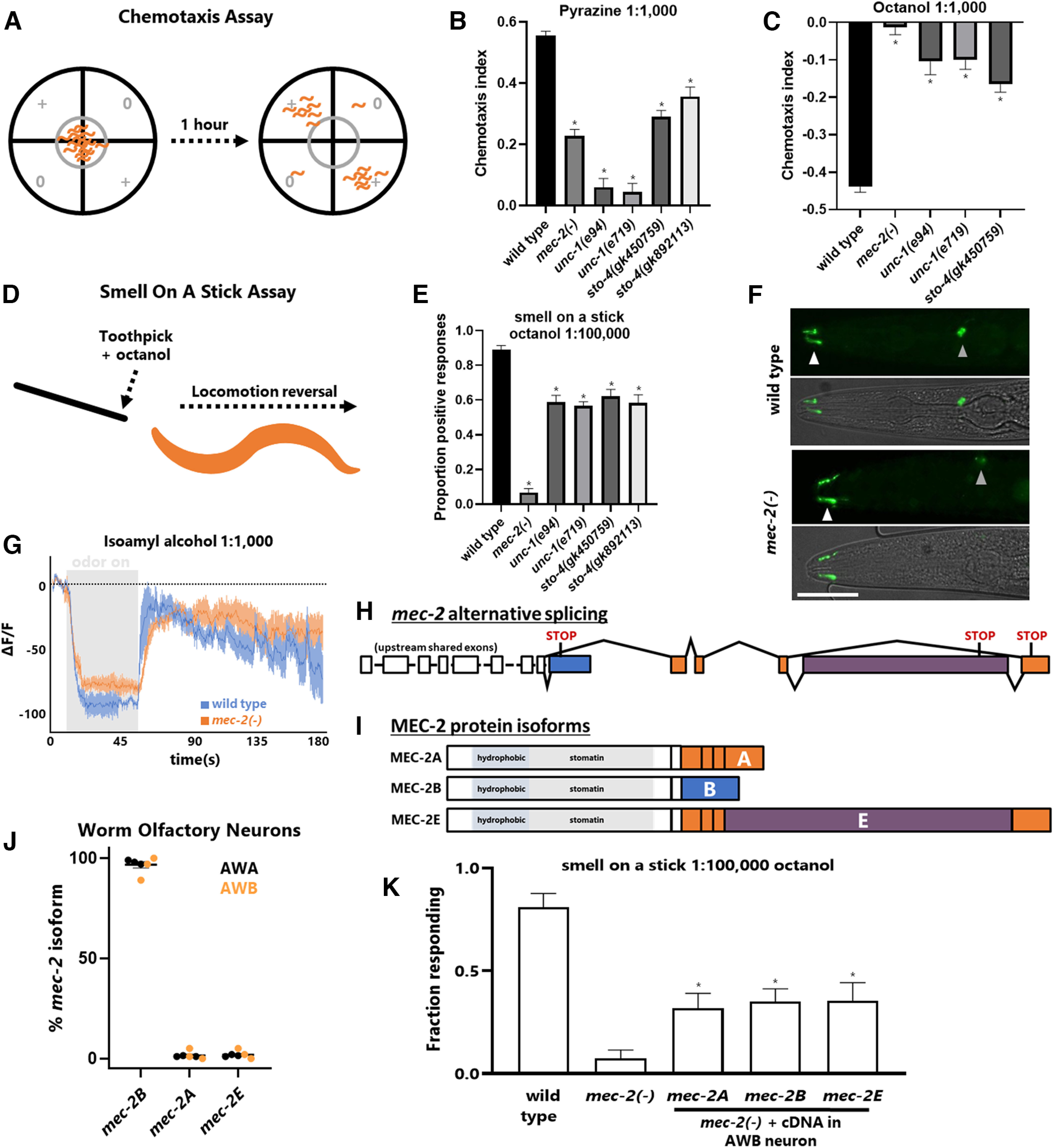
Specific stomatin domain genes required for olfaction in *C. elegans*. ***A***, Schematic of the chemotaxis assay. Worms are placed in the center of an unseeded worm plate, given a choice between point sources of odor (represented by +) and unscented diluent (represented by 0), and given 1 h to chemotax. ***B***, ***C***, Chemotaxis assays for the attractive odor pyrazine (1:1000; ***B***) or the repulsive odor octanol (1:1000; ***C***). The chemotaxis index = ((worms in + quadrant)(worms in 0 quadrant))/(worms in all quadrants), with a maximum value of 1 (attractive) and minimum value of −1 (repulsive). ***D***, Schematic of the smell on a stick assay for repulsive odors. ***E***, Smell on a stick assay for octanol. ***F***, AWA neurons labeled by translational ODR-10::GFP fusion. White arrowheads denote sensory cilia (where ODR-10 localizes). Gray arrowheads denote cell bodies. The cell body in the *mec-2* image is slightly out of the plane of focus. Scale bar represents 50 μm. See also Extended Data Figure 2-1*C*,*D*. ***G***, GCaMP5 imaging of AWC(on) neurons using a microfluidic “olfactory chip.” Isoamyl alcohol (1:1000) administration represented by the gray box. Error bars represent SEM *N* = 12 young adult worms (5 wild-type, 7 *mec-2* mutants). ***F***, Schematic of *mec-2* alternative splicing and (***G***) subsequent protein isoforms. ***H***, ***I***, *mec-2* alternative isoforms at the level of pre-mRNA splicing (***H***) and protein isoforms (***I***). The isoforms affect the divergent C terminus of *mec-2*. ***J***, CenGEN RNA Seq data reveals *mec-2* is found primarily as the *mec-2B* isoform in AWA and AWB olfactory neurons. ***K***, Smell on a stick assay, 1:100,000 octanol, statistical test (ANOVA) performed against *mec-2()* mutants. Re-expression of any *mec-2* isoform in AWB neuron partially rescues response. Asterisks indicate *p* < 0.05, one-way ANOVA. See also Extended Data Figure 2-1.

10.1523/ENEURO.0457-22.2023.f2-1 Extended Data Figure 2-1 Stomatin domain genes required for behavior in *C. elegans*. ***A***, Fraction of worms that fail to leave the 1-cm diameter origin circle after 1 h. Note that the majority of *sto-4* and *unc-1* mutants fail to locomote away from origin, whereas both wild-type and chemotaxis defective mutants do not remain in origin. ***B***, Maximal ΔF/F GCaMP signals during odor presentation of isoamyl alcohol (1:1000) as displayed in Figure 2*G*. Unpaired two-tail *t* test, *p* < 0.05. ***C***, ***D***, AWB and AWC neuronal cell bodies visualized by transgene oyIs44 (ord-1p::RFP), revealing no obvious morphological differences in cell body position, axons, or dendrites between wild-type (***C***) and *mec-2* mutant worms (***D***). Download Figure 2-1, TIF file.

Here, we show that both worms and mice express a small subset of stomatin domain genes in olfactory neurons, and that a single stomatin domain gene, *mec-2*, is strongly required for olfactory behavior in worms. We demonstrate that *mec-2* acts in a cell-autonomous manner and that multiple alternatively spliced *mec-2* isoforms can substitute for each other. We also generate a *Stoml3* knock-out (KO) mouse and demonstrate that, like its homolog *mec-2* in worms, *Stoml3* is required for proper olfactory behavior in mice. Specifically, *Stoml3* KO mice are able to detect odors, but are unable to efficiently distinguish between odors. Therefore, in addition to their conserved roles in mechanosensory behavior, *mec-2* and *Stoml3* have an additional conserved role in olfactory behavior in both worms and mice.

## Materials and Methods

### RNA Seq data

Raw fastq files were downloaded from the NCBI SRA for *C. elegans* (Taylor et al., 2021) and mouse (Bandyopadhyay et al., 2013; Kanageswaran et al., 2015) neuron-specific sequencing. Reads were mapped using STAR and alternative splicing mapped using JUM as previously reported (Dobin et al., 2013; Wang and Rio, 2018; Choudhary et al., 2021).

### Chemotaxis assays

Unseeded plates were divided into four quadrants, two with buffer and two with odorant, chemotaxis index = (# animals in two odorant quadrants)/(# animals in any of the four quadrants). Only worms which moved beyond the central circle in which they were deposited were counted. Animals were staged at larval L4 stage, M9 buffer was used for washes, animals were pelleted via. Assays were extended to 2 h to allow for a greater number of the uncoordinated worms *sto-4* and *unc-1* worms to move out of the origin. *mec-2(e75)* is used as reference allele throughout.

### Smell on a stick

Worms were staged to larval L4s. A total of 30+ worms of each genotype were transferred to unseeded plates. Each genotype was tested for lack of reaction to toothpick alone as well as toothpick and 70:30 95% EtOH: H_2_O (diluent). A 1:100,000 dilution of octanol in 70:30 95% EtOH: H_2_O was made (testing solution). A sterile toothpick was dipped into the testing solution and placed just in front of a forward moving worm. The worm was scored for positive response (moving away from the test solution) or negative response (no response). Once a worm was tested it was removed to ensure it would not be re-assayed; *n* = 30 worms per genotype per assay, assays conducted in triplicate.

### Calcium imaging

Calcium imaging was performed on young adult hermaphrodites on a microfluidic “worm chip” as previously described (Chalasani et al., 2007), using the integrated transgene kyIs722 *str-2p*::GCaMP5(D380Y) to image GCaMP in the AWC(on) neuron. Isoamyl alcohol at 1:1000 was used as olfactant.

### Mice

*Stoml3* −/− mice were generated by deleting exon 2–5 of *Stoml3* using CRISPR/Cas9 genome editing. gRNAs used to generate deletion. Upstream: CACATATGCGGGATGGTTTG and TAAACACCACATATGCGGGA. Downstream: GAGCCAAGACTCCCCAGCCC and AGTACAGCTATCCCTGGGCT. Mice were housed in a temperature and light controlled room (12/12 h dark/light cycle) and all animal experiments were conducted in accordance with policies of NIH *Guide for the Care and Use of Laboratory Animals* and Institutional Animal Care and Use Committee (IACUC). Adult mice (more than six months old) were used in this study, and both sexes of animals were used. A total of 16 wild types and 12 *Stoml3* mutants were used for each of the three olfactory tests unless otherwise noted, and the same mice were used for all behavioral tests. Behavioral videos were analyzed by an observer blind to mouse genotypes. In the block test and the habituation/dishabituation test, direct nasal contact with the odor stimulus was counted as investigation time.

### Genotyping

DNA was extracted from tails of 19- to 21-d-old mice. Wild-type and mutant mice were determined by PCR using primer set (Stoml3 common forward primer 5′–TGTTCTCCCACATGCACACC–3′, Stoml3 wild-type reverse primer 5′–GGACCCTCATTAGATGCCCC–3′, Stoml3 mutant reverse primer 5′–GGCATCAGGTCCTCTGGAAC–3′).

**Figure 3.**
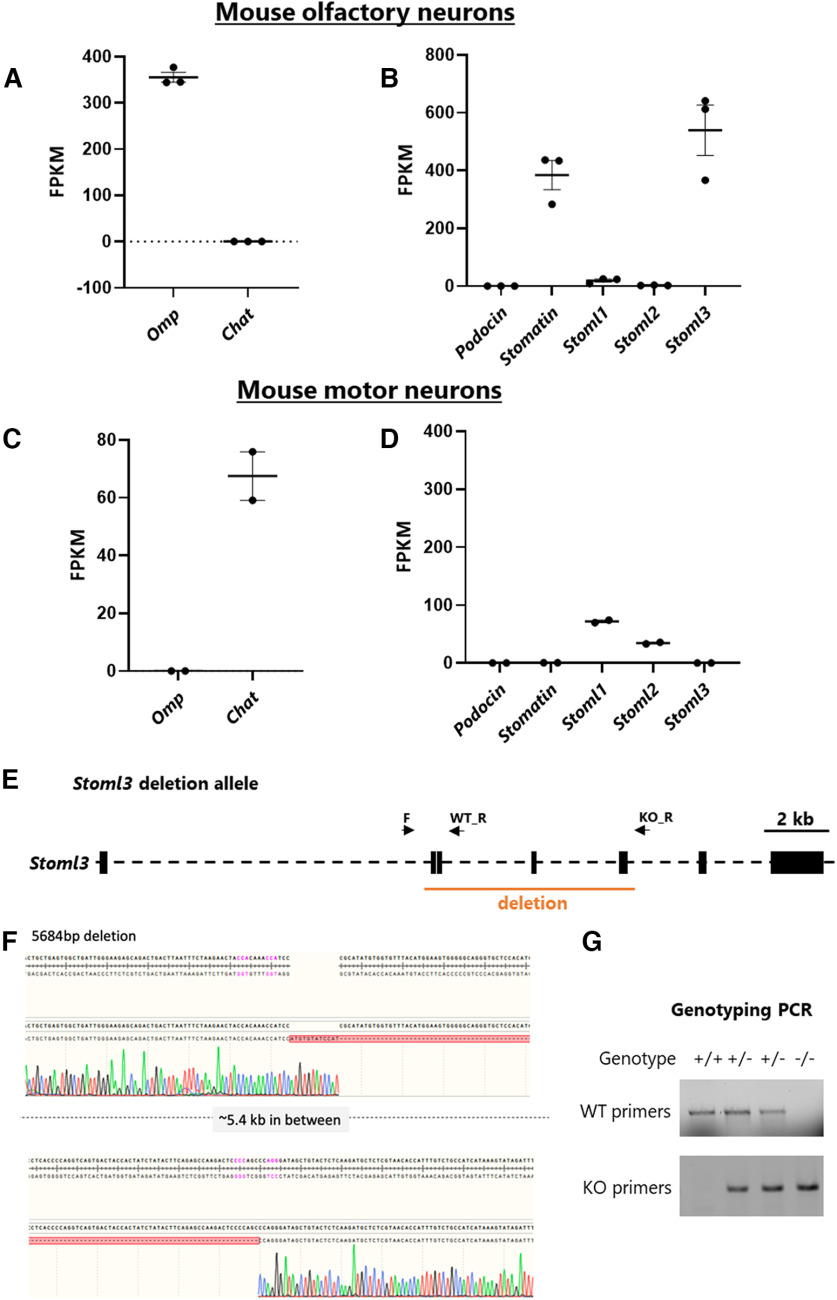
Stomatin domain genes expressed in mouse neurons, and *Stoml3* knock-out mice. ***A–D***, Cell-specific sequencing of mouse olfactory neurons (***A***, ***B***) and motor neurons (***C***, ***D***). *Omp* is used as a positive marker for olfactory neurons, and *Chat* as a positive marker for cholinergic motor neurons. ***E***, *Stoml3* gene in mouse, indicating the region we deleted in orange, and the primer sets used to detect both the wild-type and mutant genomic DNA (black arrows). ***F***, Sanger sequencing confirming the ∼5.4-kb deletion. ***G***, Genotyping gels for identifying homozygous *Stoml3* mutants and their wild-type littermates.

### Innate olfactory attraction test

Olfactory assays were conducted as previously described, with some modifications (Kobayakawa et al., 2007). Mice were isolated and habituated with a block 1 d before the test day. On the test day, mice were transferred to a clean cage with a thin layer of bedding for at least 10 min for habituation. Bedding is essential in this step to help mice reduce the fear of open space. After habituation, a scented wood block with different test odor was introduced. Animals were recorded from the front side of the cage for 3 min. Sniffing time was defined as nasal contact with the block and was measured afterward by analyzing the video. Wild-type mice (*n* = 16) and *Stoml3* knock-out mice (*n* = 11), analyzed via ordinary one-way ANOVA (*p < 0.05). Odorants used were water (control, 80 μl), peanut butter (10% w/v, 80 μl) and mouse urine (80 μl). Mouse urines were collected freshly from different litters and mixed well before use.

### Buried cereal test

Food restricted mice (90% of body weight) were used in buried cereal test to ensure mice were motivated to seek food. Mice were individually separated, and body weight was monitored every day before testing. A sweetened cereal was given to the tested mice before testing to overcome food neophobia. On all test days, mice were habituated for 1 h in their cages without water or food. Clean cages were prepared with ∼3 cm evenly distributed bedding, and one piece of sweetened cereal was buried 0.5 cm below the bedding. Mice were transferred from the habituation cage to the prepared new cage. A 5-min timer was started when the mouse was introduced in the testing cage, and time was recorded when the mouse found the cereal and began eating it. If the mouse did not uncover the pellet within 5 min, 300 s was recorded for that mouse. The buried cereal test was performed 5 d in a row, and the surface test was performed on the 6th test (cereal was put on the surface of bedding). Wild-type (*n* = 14) and *Stoml3* knock-out mice (*n* = 10). ns represents no significant difference between wild types and *Stoml3* knock-outs using Mann–Whitney *U* test. The time to uncover the buried pellet from day 3 to 5 was averaged and then compared between wild-type and mutant mice using a Mann–Whitney *U* test, similarly, time to uncover the surface pellet from 6th day was compared between groups.

### Habituation/dishabituation test

**Figure 4.**
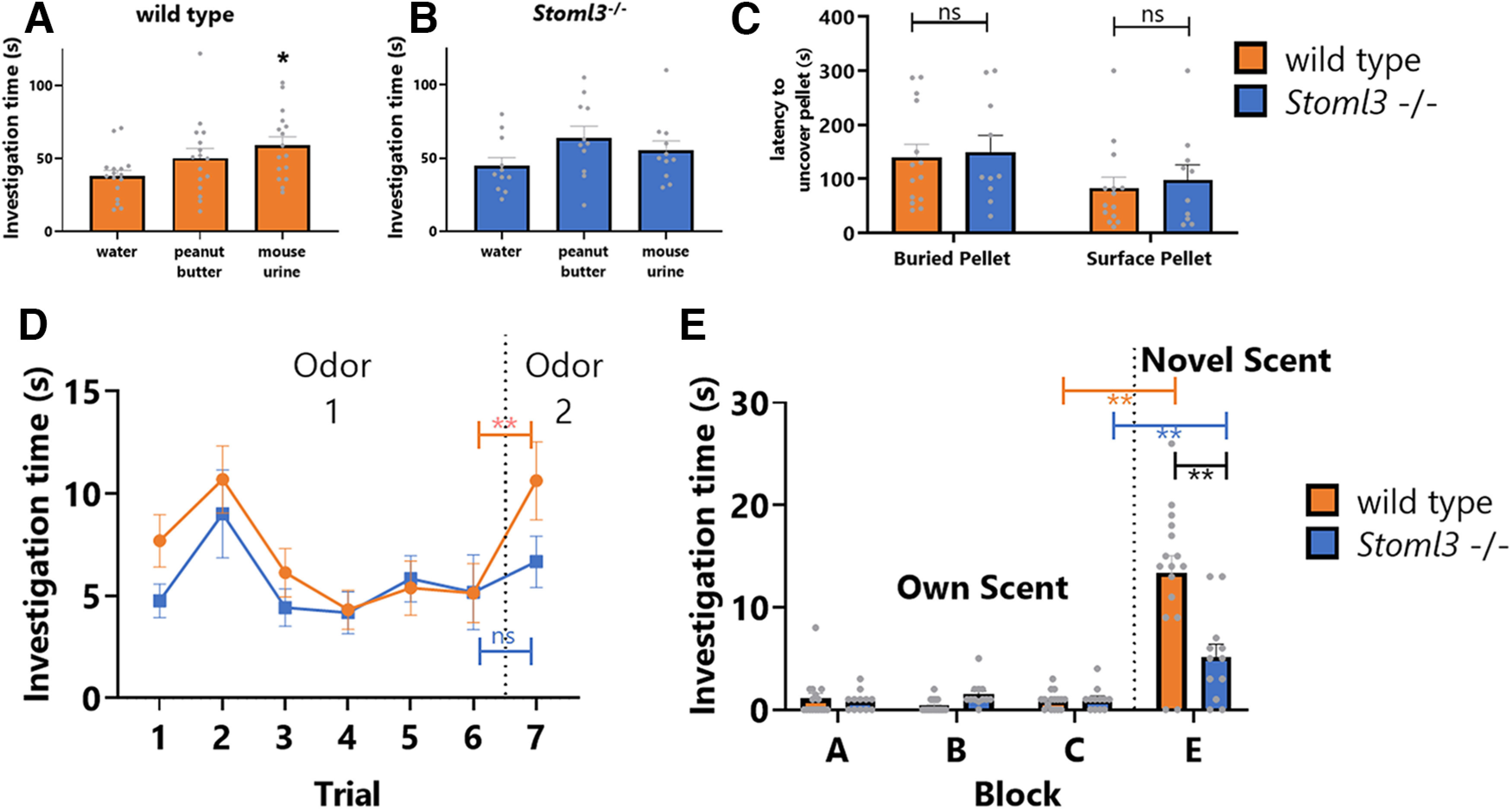
Olfactory assays for wild-type and *Stoml3* knock-out mice. ***A***, ***B***, Innate olfactory attraction tests. Mean time sniffing a scented block in wild-type mice (*n* = 16) and *Stoml3* knock-out mice (*n* = 11) during the 3-min test period. Water was used as a control scent. Peanut butter and mouse urine were used as attractive scents. Both wild-type and *Stoml3* knock-out mice showed attraction response to peanut butter and mouse urine. Asterisk indicates *p* < 0.05. ***C***, Buried cereal test. Mean time wild-type (*n* = 14) and *Stoml3* knock-out mice (*n* = 10) took to find the pellet. Surface pellet test is a positive control, confirming that the cereal pellet is attractive to the mice. ns represents no significant difference between wild types and *Stoml3* knock-outs using Mann–Whitney *U* test. ***D***, Habituation/dishabituation test. Mean time sniffing a scented cartridge in wild-type mice (*n* = 16) and *Stoml3* knock-out mice (*n* = 12) during 30-s test period across seven trials. Almond extract was used on trials 1–6, and banana extract was introduced as a novel scent on trial 7. Significant difference of sniffing time in wild types is observed when novel scent is introduced on trial 7 (**p < 0.01), while no significant difference is observed between trial 6 and trial 7 in *Stoml3* knock-out mice. ***E***, Block test. Mean time sniffing home cage blocks A–C and novel block E in wild-type mice (*n* = 16) and *Stoml3* knock-out mice (*n* = 12). Wild types spend more than 2-fold time exploring novel block (***E***) than *Stoml3* knock-out mice (**p < 0.01, Mann–Whitney *U* test, error bars represent SEM). See also Extended Data Figure 4-1.

Mice were isolated in a clean cage overnight before testing. A tissue cartridge holding a nonscented cotton ball was placed in the cage to let mice get used to the novel item. On the testing day, mice were moved to the testing area without water and food for 1-h habituation. After habituation, a scented tissue cartridge (Fig. 4*D*, noted as odor 1) was placed into cage. Animals were recorded from the front side of the cage for 30 s. This was repeated for six consecutive trials with odor 1, with intertrial intervals of 5 min. On the 7th trial, a novel odor (Fig. 4*D*, odor 2) was introduced. Time sniffing was measured on each trial during the 30-s test period. Almond extract (5 μl) was used as odor 1, banana extract (5 μl) was used as odor 2. Almond and banana scent were selected because they were considered as neutral odors for mice (Yang and Crawley, 2009). Wild-type mice (*n* = 16) and *Stoml3* knock-out mice (*n* = 12). Mean time investigating a scented cartridge during 30-s test period across seven trials. Almond extract was used on trials 1–6, and banana extract was introduced as a novel scent on trial 7. Significant difference of sniffing time in wild types is observed when novel scent is introduced on trial 7 (**p < 0.01), Mann–Whitney *U* test.

### Block test

To measure ability to discriminate social odors, we performed the block test as previously described (Lehmkuhl et al., 2014). Each mouse was individually housed in a clean cage with bedding and five wood blocks labeled A–E at least 24 h before testing. On the test day, all five blocks were removed from cage and placed into a sealed bag. Mice were transferred to testing area without food and water for 1-h habituation. After habituation, blocks A–D from the same mouse were placed back into the cage. Mice were video recorded from front side of the cage for 30 s. This procedure was repeated six times with at least 5-min interval. On the seventh trial, the same procedure was performed, but block D was replaced with block E from another mouse’s cage so that A, B, C were home-cage blocks and block E was from other mouse’s cage. Sniffing time of each block during the 30-s test period from seventh trial was measured. Wild-type (*n* = 16) and *Stoml3* knock-out mice (*n* = 12). **p < 0.01, Mann–Whitney *U* test, error bars represent SEM.

## Results

### Conserved and species-specific stomatin domain genes

We recently showed that the *C. elegans* stomatin domain protein MEC-2 is expressed in olfactory neurons in addition to its previously-described expression in mechanosensory neurons (Liang et al., 2022). We also showed that *mec-2* mutants are deficient in both mechanosensory and olfactory behaviors (Liang et al., 2022). The worm genome contains nine additional stomatin domain genes (Fig. 1*B*), and while *mec-2* has been extensively characterized in *C. elegans*, many of the other stomatin domain genes remain uncharacterized. To test whether additional stomatin domain genes are involved in olfaction, we first determined phylogenetic relationships among stomatin domain proteins in *C. elegans* and other well-studied metazoan genomes (Fig. 1*B*), with the aim of determining whether closely related genes are co-expressed in specific neuron types (Fig. 1*C*,*D*).

We noticed a striking expansion of specific stomatin domain gene members in both *Drosophila* and *C. elegans*. For example, a single *Stoml2* orthologue exists in humans, mice, flies, and worms. On the other hand, the *Stomatin*/*Stoml3* subfamily contains two genes in humans and mice, three in flies, and eight in worms (Fig. 1*B*). This observation led us to examine whether there is evidence for recent expansion of stomatin domain genes in *Drosophila* or *C. elegans*. Indeed, three of the eight *C. elegans Stomatin*/*Stoml3* homologues reside in a cluster on chromosome X (Fig. 1*E*). Such clustering is a hallmark of recently-duplicated genes (Katju and Lynch, 2003; Thomas, 2006). The remaining *C. elegans Stomatin*/*Stoml3* homologues do not reside in genomic clusters, but are all located on the X chromosome, while the more distantly-related Stomatin domain genes are distributed across autosomes.

*Drosophila* stomatin domain genes also exhibit genomic clustering: two of the three *Stomatin/Stoml3* homologues are located immediately next to each other on chromosome X (Fig. 1*F*), as are two of the three closely-related *Drosophila* homologues of *Podocin* on chromosome 3 (Extended Data Fig. 1-1*B*). Together, these results indicate that *C. elegans* and *Drosophila* genomes contain expanded repertoires of stomatin domain genes, most notably in genes related to *Stomatin*/*Stoml3*.

### Subset of stomatin domain genes expressed in *C. elegans* olfactory neurons

We analyzed RNA Seq data generated from specific FACS-sorted neuron types (Taylor et al., 2021) to determine which of the *C. elegans* stomatin domain genes are expressed in olfactory neurons. We focused on the AWA and AWB neurons, which are involved in detecting attractive and repulsive odors, respectively (Ferkey et al., 2021). AWA and AWB cell bodies are located near the terminal pharyngeal bulb. Their dendrites extend anteriorly to the nose tip, ending in sensory cilia, and their axons synapse onto downstream interneurons. It was previously shown that *mec-2* is required for detection of both AWA-specific and AWB-specific odorants (Liang et al., 2022), and cell-specific RNA Seq confirms that *mec-2* is highly expressed in both AWA and AWB neurons (Fig. 1*C*; Extended Data Fig. 1-1*A*). As a comparison, we also assayed expression of stomatin domain genes in two classes of neurons that are not sensory neurons, the VD and DD motor neurons. In contrast to olfactory neurons, *mec-2* expression is nearly undetectable in motor neurons (Fig. 1*D*). This demonstrates that *mec-2* is expressed in specific subsets of neurons, including the AWA and AWB olfactory neurons.

We also detected substantial levels of expression for both *sto-4* and *unc-1* in AWA and AWB olfactory neurons (Fig. 1*C*). In contrast to *mec-2*, however, *sto-4* and *unc-1* are also highly expressed in motor neurons, suggestive of a broader function in various neuron types (Fig. 1*D*). Indeed, using both transcriptional and translational reporters, *unc-1* was previously shown to be expressed and active in a variety of muscles and neurons, including motor neurons (Chen et al., 2007; Rajaram et al., 1999). Finally, we note that within the functional classes of neurons we tested, stomatin domain genes tend to exhibit similar expression levels between individual cell sub-types (e.g., mec-2 is highly expressed in both AWA and AWB). The one exception is *sto-2*, which is highly expressed in motor neurons, but with consistent >3-fold higher expression in DD than in VD motor neurons (Fig. 1*D*). Together, these data reveal a diversity of stomatin domain genes in the *C. elegans* genome, which exhibit cell-type specific gene expression within the nervous system.

### Specific stomatin domain genes required for *C. elegans* olfactory behavior

We next tested whether the Stomatin domain genes expressed in olfactory neurons are required for olfactory behavior. Using standard chemotaxis assays (Fig. 2*A*) we previously showed that *mec-2* is required for chemotaxis responses to a variety of volatile odorants, both attractive and repulsive (Liang et al., 2022; Fig. 2*B*,*C*). We obtained multiple loss-of-function alleles for both of the olfactory-neuron-expressed stomatin domain genes, *unc-1* and *sto-4*. We found that mutations in both genes cause reduced locomotion, complicating the interpretation of standard chemotaxis assays, which require coordinated locomotion over time toward or away from the olfactants. Only worms that move from the origin in which they are deposited (Fig. 2*A*) are assayed, to control for animals that are unable to locomote. The majority of *unc-1* and *sto-4* animals remain within the origin, in contrast to wild types or *mec-2*, indicating an inability to locomote effectively (Extended Data Fig. 2-1*A*). Even so, among the minority of animals locomoting beyond the origin, both *unc-1* and *sto-4* mutants exhibit chemotaxis defects (Fig. 2*B*,*C*), although we reasoned that this could still be attributable to locomotory deficits.

To further disentangle the effects of *unc-1* and *sto-4* on olfaction versus locomotion, we turned to a “smell on a stick” assay, in which individual worms are directly presented with an aversive odor, causing wild-type worms to quickly perform a locomotion reversal to avoid the odor (Fig. 2*D*). This assay requires much less coordination of locomotion, allowing us to focus on the olfactory abilities of the mutants. *mec-2* mutants exhibit strong defects in this assay when presented with the repellant odor octanol, while *sto-4* and *unc-1* mutations cause only minor olfactory defects (Fig. 2*E*). From these results, we conclude that *mec-2* is the primary stomatin domain gene required for olfaction in *C. elegans*. While *sto-4* and *unc-1* are expressed in olfactory neurons, they seem to play a minor role in olfaction itself, and their requirement for chemotaxis stems largely from locomotory defects. This is consistent with their high levels of expression in motor neurons as well (Fig. 1*C*).

In mechanosensation, *mec-2* has been shown to affect activity of mechanosensory channels, but not the development or structure of the mechanosensory neurons themselves (Zhang et al., 2004). We likewise wanted to test whether *mec-2* plays a role in the development or activity of olfactory neurons. We examined the structure of the AWA neuron and found no differences between wild-type and *mec-2* mutants: their cell bodies, dendrites, and cilia are all positioned appropriately, as is the olfactory receptor ODR-10 (Fig. 2*F*). We likewise found no morphologic differences in AWB or AWC neurons (Extended Data Fig. 2-1*C*,*D*) We therefore conclude that *mec-2* is not required for the development or morphology of the AWA olfactory neuron.

We also tested the activity of olfactory neurons in wild-type and *mec-2* animals, using the well-characterized *str-2::GCaMP5* reporter of calcium activity in AWC(on) olfactory neurons (Chalasani et al., 2007; Akerboom et al., 2012). As previously described, in wild-type neurons odor presentation results in a robust decrease in calcium, and odor removal results in a robust increase in calcium (Chalasani et al., 2007; Akerboom et al., 2012; Fig. 2*G*). In *mec-2* mutants, the calcium response is broadly similar, but the magnitude of the calcium response is decreased (Fig. 2*G*). The magnitude of the decrease is modest, but statistically significant (Extended Data Fig. 2-1*B*). From these experiments we conclude that *mec-2* has a modest effect on olfactory neuron activity in response to olfactory stimuli.

Finally, to test whether *mec-2* acts cell autonomously in olfactory neurons, we performed the smell on a stick assay in *mec-2* mutants with cell-specific *mec-2* re-expression. We used the *str-1* promoter to re-introduce *mec-2* to the AWB neuron, which is involved in sensing the aversive odor octanol (Chao et al., 2004). We previously demonstrated that *mec-2* encodes three different isoforms with distinct functional properties in different cell types (Fig. 2*H*,*I*). In agreement with previous work using reporters (Liang et al., 2022), cell-specific RNA Seq indicates that the *mec-2B* isoform predominates in AWA/AWB olfactory neurons (Fig. 2*J*). We generated cDNAs for each of the three isoforms and tested whether each could rescue the olfactory response to octanol when artificially expressed in AWB. Indeed, transgenic re-introduction of any of the three *mec-2* isoforms in the AWB neuron partially rescues octanol avoidance responses in *mec-2* mutants (Fig. 2*K*). This is in contrast with previous work in mechanosensory neurons, which showed that *mec-2B* is not functional, while *mec-2A* and *mec-2E* must be co-expressed for proper mechanosensory behavior (Liang et al., 2022). Together, these experiments indicate that all three *mec-2* isoforms are functional when ectopically expressed in AWB, and that *mec-2* acts cell-autonomously in the AWB olfactory neuron.

### Subset of stomatin domain genes expressed in mouse olfactory neurons

Previous work in *C. elegans* demonstrated a critical role for *mec-2* in mechanosensory behavior, and inspired follow-up work in mice demonstrating a role for *Stoml3* in mammalian mechanosensory behavior (Huang et al., 1995; Wetzel et al., 2007). We similarly wished to determine whether the role for *mec-2* in olfaction represents a novel evolutionarily-conserved function for stomatin domain genes.

We first tested whether specific stomatin domain genes are selectively expressed in mouse olfactory receptor neurons (ORNs). To do so we analyzed RNA Seq data in which mouse olfactory receptor neurons were GFP labeled and FACS sorted, and compared this with similar data obtained from mouse motor neurons (Bandyopadhyay et al., 2013; Kanageswaran et al., 2015; Fig. 3*A–D*). We confirmed that marker genes for the respective neuron classes are expressed in the expected cell types (Fig. 3*A*,*C*), and found that stomatin domain genes exhibit distinct expression patterns (Fig. 3*B*,*D*). We observed high expression of both *Stoml3* and *Stomatin* in ORNs, but not in motor neurons, while *Stoml1* and *Stoml2* are expressed in motor neurons but not olfactory neurons (Fig. 3*A*,*B*). Because *Stoml3* displays the highest level of expression in ORNs, and because of previous work demonstrating striking localization of *Stoml3* to ORN sensory cilia (Kobayakawa et al., 2002; Goldstein et al., 2003), we prioritized *Stoml3* for further genetic and behavioral analysis.

### *Stoml3* is required for a subset of olfactory behaviors in mice

*Stoml3* KO mice have previously been generated (Wetzel et al., 2007), but we were unable to obtain these mice, so we used CRISPR/Cas9 to generate a similar *Stoml3* KO mouse (Fig. 3*E–G*). We removed the same two exons as in the published *Stoml3* KO, as well as two additional downstream exons. The deletion results in the loss of the conserved stomatin domain of *Stoml3* and introduction of downstream premature stop codons. We found that *Stoml3*−/− mice are born at Mendelian ratios and do not display overt defects in development, behavior, grooming, or body weight. This is in agreement with the previously-generated *Stoml3*−/− mice, which were further shown to possess normal motor performance (Wetzel et al., 2007).

To test whether *Stoml3* mutant mice are defective in olfactory behavior, we performed a number of standard olfactory behavioral assays on *Stoml3*−/− mice and their wild-type littermates. We first tested the ability to detect and respond to olfactory stimuli that elicit innate behavioral responses in wild-type mice (Kobayakawa et al., 2007; Qiu et al., 2021). We measured time spent sniffing attractive odors compared with controls and found that both wild-type and *Stoml3* mutant mice tend to spend more time sniffing attractive odors, although the trend only reaches statistical significance for urine scent in wild type (Fig. 4*A*,*B*). Time spent investigating each scent does not significantly differ between wild-type and *Stoml3* mutants (Extended Data Fig. 4-1*A*), suggesting that *Stoml3* mutants are not grossly different from wild type in their ability detect odors. We next measured latency to find and eat a buried cereal pellet, because mice with impaired olfaction have been demonstrated to take longer to uncover buried food (Lehmkuhl et al., 2014; Nathan et al., 2004). As with the innate olfactory assays, we found no differences between *Stoml3* and wild-type mice in latency to uncover the buried pellet (Fig. 4*C*, S3B, *p* > 0.01). Together, these results suggest that loss of *Stoml3* does not strongly affect innate olfactory response to attractive stimuli such as food odors.

10.1523/ENEURO.0457-22.2023.f4-1 Extended Data Figure 4-1 *Stoml3* behavior and *mec-2* expression. ***A***, Latency to uncover pellet in cereal test measured on a daily basis, showing no significant differences between dynamics of wild-type and *Stoml3* KO mice. ***B***, In trial 6 of the block test, where no novel odor is present, time spent exploring the blocks is minimal. ***C***, Single-cell sequencing data from CenGEN consortium data on *mec-2* expression in representative neuron populations. Size of dot represents proportion of single cells in which *mec-2* was detected, and heatmap represents scaled TPM of the *mec-2* gene. A few sensory cell types are highlighted. Download Figure 4-1, TIF file.

To test whether *Stoml3* knock-out mice are able to distinguish between different odors, we performed a habituation/dishabituation test in which mice are habituated with one scent for six consecutive trials, then switched to a novel scent on the seventh trial (Lehmkuhl et al., 2014). We measured the time spent sniffing the odor cartridge during each trial. Both wild-type and *Stoml3* mutant mice display an initial interest in the odor followed by a period of habituation and decreased interest in the odor, as previously described (Lehmkuhl et al., 2014). On the seventh trial, the odor was changed to a novel scent. Wild-type mice spend more time sniffing the novel scent compared with a familiar scent (Fig. 4*D*, *p* < 0.01), as previously described (Wesson et al., 2008). In contrast, *Stoml3* mutant mice do not exhibit an increased interest in the novel scent in the seventh trial (*p* > 0.01) and spend the same amount of time investigating the novel scent in trial 7 as with the habituated scent in trial 6. This suggests that *Stoml3* mice, unlike wild types, are unable to (or unmotivated to) discriminate between familiar and novel scents (Fig. 4*D*).

Further evidence for this hypothesis comes from experiments on social odors in the “block test.” In this test, we housed mice individually with several wooden blocks for 24 h, allowing the blocks to acquire the odors of the home cage (Lehmkuhl et al., 2014). The next day, we removed and reintroduced these home cage blocks for six consecutive trials to measure investigation time of the familiar blocks. Mice became habituated to the presence of the blocks and spent minimal time investigating them by the sixth trial (Extended Data Fig. 4-1*C*). On the seventh trial, one of the blocks was swapped with a block from the cage of a different mouse, thus introducing a block with novel social odors. Wild-type mice spend a substantial amount of time sniffing of the novel-odor block, as previously described (Wesson et al., 2008; Fig. 4*E*, *p* < 0.01). *Stoml3* mutants also spend more time sniffing the novel-odor block, but spend significantly less time sniffing the novel-odor block when compared with wild-type mice (Fig. 4*E*, *p* < 0.01). This result indicates a deficit in responding to self versus nonself social odors, which could arise from reduced interest/motivation, discriminatory ability, or a combination of factors.

Together, these experiments demonstrate that *Stoml3* knock-out mice do not completely lose the ability to detect odors (Fig. 4*A–C*). Rather, loss of *Stoml3* impairs the behavioral response to odor discrimination, including familiar versus novel odors (Fig. 4*D*), and self versus nonself odors (Fig. 4*E*).

## Discussion

Here, we demonstrate that worms and mice express a small subset of stomatin domain genes in olfactory neurons. Of these, *mec-2* and *Stoml3* are required for proper olfactory behavior in both worms and mice. In worms, *mec-2* is required for chemotaxis, acts in a cell-autonomous manner, and multiple alternatively spliced *mec-2* isoforms can substitute for one another. In mice, we find *Stoml3* to be highly expressed in olfactory neurons. Our trancscriptomic analysis agrees with previous work showing that *Stoml3* expression is highly restricted (Kobayakawa et al., 2002; Goldstein et al., 2003). It is undetectable in most tissues, but strongly expressed in olfactory neurons and in mechanosensory neurons of the dorsal root ganglia (Goldstein et al., 2003; Wetzel et al., 2007). Given this highly restricted expression, we speculate that in mice, as in worms, the function of *Stoml3* in olfactory behavior is cell-autonomous. We show that *Stoml3* KO mice are able to detect odors, but unable to efficiently distinguish between odors. Together, these experiments suggest that in addition to their well-established roles in mechanosensory behavior, *mec-2* and *Stoml3* have an additional shared role in olfactory behavior.

The precise biochemical role of stomatin domain proteins remains somewhat mysterious. A common theme is the modulation of ion channel function (Price et al., 2004; Huber et al., 2006), but the molecular mechanisms have yet to be fully elucidated. A model consistent with our data are that *mec-2* and *Stoml3* modulate ion channel activity in olfactory neurons in a manner required for their normal levels of activity. It will be interesting to test whether *mec-2/Stoml3* exhibit similar or distinct interactions with ion channels or other membrane proteins in mechanosensory versus olfactory neurons. In a recent study, electrophysiological recordings from olfactory epithelium slices revealed that *Stoml3* KO ORNs exhibit modest reductions in spontaneous firing frequency (Agostinelli et al., 2021, p. 3). Odor-evoked firing frequency is not affected, but spike duration and number are modestly reduced (Agostinelli et al., 2021, p. 3). These electrophysiological properties of *Stoml3* KO OSNs could underly the deficits in olfactory behavior we describe here.

We now know that *mec-2* and *Stoml3* are required for at least two different sensory behaviors mediated by two different sensory neuron types (mechanosensory and olfactory). Are there additional neuronal roles yet to be uncovered? We note that in *C. elegans*, *mec-2* transcripts are expressed not only in the AWA and AWB olfactory neurons described here, but at comparably high levels in gustatory, thermosensory, and other specific neuronal types as well (Extended Data Fig. 4-1*D*). It will be interesting to determine whether *mec-2* and/or *Stoml3* also play a role in these sensory activities as they do in mechanosensation and olfaction.

## Synthesis

Reviewing Editor: Christina Zelano, Northwestern University - Chicago Decisions are customarily a result of the Reviewing Editor and the peer reviewers coming together and discussing their recommendations until a consensus is reached. When revisions are invited, a fact-based synthesis statement explaining their decision and outlining what is needed to prepare a revision will be listed below. The following reviewer(s) agreed to reveal their identity: Keerthi Krishnan, Adam Dewan.

Reviewers agreed that this manuscript is potentially significant and impactful, and will be of interest to a wide audience. It presents comparable findings across two different species on a novel role for a gene family. Both reviewers raised important concerns that need to be addressed:

Reviewer 1:

The manuscript titled “A conserved role for stomatin domain genes in olfactory behavior” characterizes a new role for mec-2/Stoml3 gene in olfactory behavior. The comparative approach of using C. elegans and mice is a definite plus, in uncovering a novel role, in this study. This reviewer would encourage the authors of the study to clarify/elaborate on the following points, and provide stronger rationale for the explanation of some interpretations in the study.

1. Page 5, line 104 and Figure 1C: mec-2 is considered as “highly expressed in both AWA and AWB neurons”. What is a typical number of FKPM to be considered “highly expressed?” A positive control for high expression or already published reports for high expression would be helpful.

2. Are mRNA levels congruent with protein levels for these genes in C. elegans?

3. Page 5, line 105: What is the connection between AWA, AWB, VD and DD neurons? Are they from the same lineage? Or, work in a circuit together? Why are the VD and DD neurons used for comparison? Please explain.

4. Figure 2K - Is it feasible to “rescue” with double or triple mec-2 isoform re-expressions? Is this a technically difficult experiment to perform?

5. Pg. 10: Where else is Stoml3 RNA and protein expressed in the mouse? Is it possible that Stoml3 is not only expressed in olfactory neurons, while interpreting the olfactory-motor assays? Please discuss.

6. Figure 4. Were the same mice used for all the olfactory assays in a battery of behavioral tests? Please clarify in the “Methods”. A scatter plot would be more useful graphical representations, to see individual variation and response to different stimuli. In Figure 4D, what is the explanation for the rise and dip in time sniffing for both WT and KO between days 1-3? Also, for interpretation of the Results here, is it possible that the Stoml3KO do not care or are not interested in olfactory discrimination or lack of preference between scents, rather than have “impaired ability to distinguish”? Please discuss.

7. Page 15: In the “buried cereal test”, is there a rationale for averaging time across days? The dynamics in unearthing the buried cereal over days would be useful. For example, do the WT get faster each day? Do the KO show similar time every day?

Reviewer 2:

This manuscript provides evidence for a role for stomatin genes in olfactory behavior. This conclusion is not surprising given STOML-3 is expressed in cilia of olfactory sensory neurons and its genetic removal alters transduction (Agostinelli et al, 2021). However, the data from C. elegans and the behavior experiments in mice are interesting and would provide further support for the contribution of this gene to olfactory perception. Thus, I feel that the topic is significant and would be impactful to a broad audience.

I could not find the statistical analysis for the odor attraction assay. The authors have compared the wrong data in the block experiment.

This manuscript provides evidence for a role for stomatin genes in olfactory behavior. This conclusion is not surprising given STOML-3 is expressed in cilia of olfactory sensory neurons and its genetic removal alters transduction (Agostinelli et al, 2021). However, the data from C. elegans and the behavior experiments in mice are interesting and would provide further support for the contribution of this gene to olfactory perception. Thus, I feel that the topic is significant and would be impactful to a broad audience. However, there are several prominent issues with their behavioral experiments in mice that I hope the authors can address.

1. Since mec-2 affect the activity of the mechanosensory channels, could the authors provide evidence that mec-2 knockout worms exhibit normal locomotor activity? Further, the authors also state that unc-1 and sto-4 mutants exhibit reduced locomotion, so a panel showing overall locomotor ability compared to a wt would help support the narrative.

2. Could the authors please explain why morphology was examined in the AWA neuron only, while transduction was only tested in AWC neurons? What about AWB neurons? Further, since this paper could have a broad audience a very brief description of the olfactory circuitry of C. elegans would be helpful.

3. The authors conclude that mec-2 causes a small, but statistically significant decrease in calcium response to isoamyl acetate. Could the authors provide more data on the number of worms tested? Further, this decrease in olfactory response would be better supported with a second odor. However, I do not deem this additional experiment necessary.

4. I have several concerns about the behavioral experiments in mice.

Major 1: In the habituation dishabituation assay, why is there a noticeable increase in the second presentation of the odor? This is highly unusual. An animal should initially investigate a novel odor for the greatest time period upon the first presentation and then subsequently decrease their investigation. This is a very key point because without the second presentation (which is unusual) the investigation of odor 1 and 2 by the mutant animal is flat. This would denote an overall lack of interest in the odor/task (or another confounding issue) not necessarily a discrimination deficit.

Major 2: The authors have incorrectly interpreted their block experiment. A significant change in investigation time between two presentations denotes discrimination, not a difference between the investigation time between two genotypes. Thus, the mutant mice appear to be able to discriminate between a novel scent and their own scent as the statistical comparison would appear to be significant between blocks C and E. The difference between the investigation time between the wild types and mutants denotes a difference of interest (not discrimination), which again could be due to any number of reasons.

Major 3: Is the investigation time for peanut butter and mouse urine significantly different from water? I don’t see any statistical tests for this comparison. This is another key point for the authors conclusions.

Minor 1: Could the authors please provide more details about the analysis of this data? Was the data analyzed by an observer blind to genotype etc? Did the authors measure nasal contact or just X distance from the source? Some of these details are included for the innate attraction test but are missing from the other behavioral assays. The number of animals should be included in the behavioral descriptions.

Minor 2: The Y axis for Figure 4 should be investigation time, not sniffing. Mice are also sniffing when they are not near the stimulus. The error bars are not mentioned - are they stdev? For panels A,B,C, and E individual data points would be more impactful.

Minor 3: Given the widespread expression of stoml3, could the authors provide some behavioral evidence that these animals are relatively normal.

Minor Concerns

Line 30 - FYI - more recent work has called into question the ability of humans to discriminate 1 trillion olfactory stimuli. Gerkin and Castro, 2015 eLife and others.

Line 134 - Although I don’t deem this experiment necessary - I wonder whether the authors could provide more evidence for or against the minimal contribution of unc-1 and sto-4 for olfactory behavior by performing an analogous experiment to “smell the stick” with a non-olfactory stimulus (e.g., heat or light?). A similar reduction in locomotion would support that these genes are making a larger contribution to locomotor ability, while a similar locomotor response to wts could provide evidence for a role in olfactory behavior.

## Author Response

We thank the reviewers for their useful comments and suggestions. We have accordingly performed a number of additional experiments and analyses, and altered and/or elaborated on a number of interpretations and conclusions. We believe these changes enhance the methods presentation, data analysis, and data interpretation. Newly-added text in the manuscript, and response to reviewers here, are denoted by blue font.

Synthesis Statement for Author (Required):

Reviewers agreed that this manuscript is potentially significant and impactful, and will be of interest to a wide audience. It presents comparable findings across two different species on a novel role for a gene family. Both reviewers raised important concerns that need to be addressed:

Reviewer 1:

The manuscript titled “A conserved role for stomatin domain genes in olfactory behavior” characterizes a new role for mec-2/Stoml3 gene in olfactory behavior. The comparative approach of using C. elegans and mice is a definite plus, in uncovering a novel role, in this study. This reviewer would encourage the authors of the study to clarify/elaborate on the following points, and provide stronger rationale for the explanation of some interpretations in the study.

1. Page 5, line 104 and Figure 1C: mec-2 is considered as “highly expressed in both AWA and AWB neurons”. What is a typical number of FKPM to be considered “highly expressed?” A positive control for high expression or already published reports for high expression would be helpful.

We have now added a positive control: osm-6, which is often used as a marker of ciliated sensory neurons (including olfactory neurons). We detect expression of osm-6 in olfactory neurons but not motor neurons as expected (Fig S1A), and the FPKM values are on par with those of the Stomatin domain genes tested in olfactory neurons.

2. Are mRNA levels congruent with protein levels for these genes in C. elegans?

We have now added text discussing that for both unc-1 and mec-2, gene-specific translational reporters are congruent with the mRNA levels tested here (lines 60-62 and 113). For sto-4 and other genes, this remains to be determined.

3. Page 5, line 105: What is the connection between AWA, AWB, VD and DD neurons? Are they from the same lineage? Or, work in a circuit together? Why are the VD and DD neurons used for comparison? Please explain.

We have now added a brief explanation as to our rationale in choosing which neurons to examine (lines 106-108). The main goal was to test olfactory sensory neurons (AWA and AWB) and compare them to control neuron types that are not sensory neurons. Deep single-cell transcriptome data are currently only available for a handful of neurons, restricting our possible choices, and we settled on olfactory neurons versus motor neurons. We also felt this would provide a nice parallel to available cell-specific sequencing data from mouse, in which olfactory and motor neuron RNA Seq data is available.

4. Figure 2K - Is it feasible to “rescue” with double or triple mec-2 isoform re-expressions? Is this a technically difficult experiment to perform?

This experiment would be modestly difficult to perform, but possible. We speculate, however, that the reason for only partial rescue with each cDNA is not that all three isoforms are simultaneously required, but rather that ectopic transgenic rescue is not fully efficient. We suspect this because (1) The AWB neuron normally expresses only a single mec-2 isoform, and (2) we previously showed that endogenous editing of the genome (as opposed to transgenic overexpression) to provide only the mec-2B isoform results in nearly 100% rescue.

5. Pg. 10: Where else is Stoml3 RNA and protein expressed in the mouse? Is it possible that Stoml3 is not only expressed in olfactory neurons, while interpreting the olfactory-motor assays? Please discuss.

We have now added some discussion on this point, including that Stoml3 has previously been shown (and in agreement with our transcriptomic data) to be highly restricted in expression, detectable specifically in olfactory neurons and mechanosensory neurons of the DRG. Which suggests that the olfactory behavior defects in mice, as demonstrated in worms, are likely (though not 100% conclusively) to be cell-autonomous in nature (lines 290-295).

6. Figure 4. Were the same mice used for all the olfactory assays in a battery of behavioral tests? Please clarify in the “Methods”.

We have now added this information in the Methods section, that the same mice were used across all behavioral assays.

A scatter plot would be more useful graphical representations, to see individual variation and response to different stimuli.

We have now added individual data points overlaid on these graphs.

In Figure 4D, what is the explanation for the rise and dip in time sniffing for both WT and KO between days 1-3? Also, for interpretation of the Results here, is it possible that the Stoml3KO do not care or are not interested in olfactory discrimination or lack of preference between scents, rather than have “impaired ability to distinguish”? Please discuss.

We speculate that this phenomenon is due to the fact that each trial was allotted 30 seconds (as in PMID 25177842) as opposed to 2 minutes (as in PMID 19575474 17991495 and others), and the fact that mice were housed 24 hours prior to testing (as in PMID 25177842 17623023) rather than being transferred to fresh cages prior to testing (as in PMID 9575474 34458470). Together we believe this resulted in a delay for the mice (both wild-type and mutant) to begin investigating on the 1st trial. We noticed anecdotally in reviewing the behavioral videos that mice took longer to begin investigation during the 1st trial than in the 2nd trial. However, once the mice began to investigate during the 1st trial, they continued to investigate robustly. As such, we speculate that if the trials were designed for longer duration, the investigation time of the 1st trial would equal or surpass that of the 2nd trial.

Importantly, the increase in investigation time from trial 1 to trial 2 is seen in both wild-type controls as well as Stoml3 mutants, and therefore should not alter the conclusion that wild-types strongly dishabituate from trial 6 to 7, while Stoml3-/- do not. Nevertheless, we have also added some additional discussion as to the possibility that Stoml3 mice could be uninterested, as opposed to unable, to distinguish and respond to olfactory cues (lines 252-253 and 264-265).

7. Page 15: In the “buried cereal test”, is there a rationale for averaging time across days? The dynamics in unearthing the buried cereal over days would be useful. For example, do the WT get faster each day? Do the KO show similar time every day?

We now present both the original averaged data, and also the dynamics (Fig 4C and S4B). We did not identify any significant differences between wild-type and KO. There is a trend, but not statistically significant, toward faster unearthing of the pellet over time in WT.

Reviewer 2:

This manuscript provides evidence for a role for stomatin genes in olfactory behavior. This conclusion is not surprising given STOML-3 is expressed in cilia of olfactory sensory neurons and its genetic removal alters transduction (Agostinelli et al, 2021). However, the data from C. elegans and the behavior experiments in mice are interesting and would provide further support for the contribution of this gene to olfactory perception. Thus, I feel that the topic is significant and would be impactful to a broad audience.

I could not find the statistical analysis for the odor attraction assay. The authors have compared the wrong data in the block experiment.

This manuscript provides evidence for a role for stomatin genes in olfactory behavior. This conclusion is not surprising given STOML-3 is expressed in cilia of olfactory sensory neurons and its genetic removal alters transduction (Agostinelli et al, 2021). However, the data from C. elegans and the behavior experiments in mice are interesting and would provide further support for the contribution of this gene to olfactory perception. Thus, I feel that the topic is significant and would be impactful to a broad audience. However, there are several prominent issues with their behavioral experiments in mice that I hope the authors can address.

1. Since mec-2 affect the activity of the mechanosensory channels, could the authors provide evidence that mec-2 knockout worms exhibit normal locomotor activity? Further, the authors also state that unc-1 and sto-4 mutants exhibit reduced locomotion, so a panel showing overall locomotor ability compared to a wt would help support the narrative.

We have now added a panel specifically measuring locomotory ability (Fig S2A) which shows poor locomotory ability in unc-1 and sto-4 mutants compared to wild type, and better locomotory ability in the mec-2 and chemotaxis-negative che-2 mutants.

2. Could the authors please explain why morphology was examined in the AWA neuron only, while transduction was only tested in AWC neurons? What about AWB neurons? Further, since this paper could have a broad audience a very brief description of the olfactory circuitry of C. elegans would be helpful.

We have now added a brief description of the olfactory circuitry of C. elegans (lines 100-105). We have also now added a new experiment in which we examined morphology of AWB and AWC neurons as well (Figure S2C-D) and find no obvious morphological defects in mec-2 mutants, similar to our findings in the AWA neuron.

3. The authors conclude that mec-2 causes a small, but statistically significant decrease in calcium response to isoamyl acetate. Could the authors provide more data on the number of worms tested? Further, this decrease in olfactory response would be better supported with a second odor. However, I do not deem this additional experiment necessary.

We have now added this information in the figure legend (Figure 2G legend).

4. I have several concerns about the behavioral experiments in mice.

Major 1: In the habituation dishabituation assay, why is there a noticeable increase in the second presentation of the odor? This is highly unusual. An animal should initially investigate a novel odor for the greatest time period upon the first presentation and then subsequently decrease their investigation. This is a very key point because without the second presentation (which is unusual) the investigation of odor 1 and 2 by the mutant animal is flat. This would denote an overall lack of interest in the odor/task (or another confounding issue) not necessarily a discrimination deficit.

[Same text as response to Reviewer 1, above]: We speculate that this phenomenon is due to the fact that each trial was allotted 30 seconds (as in PMID 25177842) as opposed to 2 minutes (as in PMID 19575474 17991495 and others), and the fact that mice were housed 24 hours prior to testing (as in PMID 25177842 17623023) rather than being transferred to fresh cages prior to testing (as in PMID 9575474 34458470). Together we believe this resulted in a delay for the mice (both wild-type and mutant) to begin investigating on the 1st trial. We noticed anecdotally in reviewing the behavioral videos that mice took longer to begin investigation during the 1st trial than in the 2nd trial. However, once the mice began to investigate during the 1st trial, they continued to investigate robustly. As such, we speculate that if the trials were designed for longer duration, the investigation time of the 1st trial would equal or surpass that of the 2nd trial.

Importantly, the increase in investigation time from trial 1 to trial 2 is seen in both wild-type controls as well as Stoml3 mutants, and therefore should not alter the conclusion that wild-types strongly dishabituate from trial 6 to 7, while Stoml3-/- do not. Nevertheless, we have also added some additional discussion as to the possibility that Stoml3 mice could be uninterested, as opposed to unable, to distinguish and respond to olfactory cues (lines 252-253 and 264-265).

Major 2: The authors have incorrectly interpreted their block experiment. A significant change in investigation time between two presentations denotes discrimination, not a difference between the investigation time between two genotypes. Thus, the mutant mice appear to be able to discriminate between a novel scent and their own scent as the statistical comparison would appear to be significant between blocks C and E. The difference between the investigation time between the wild types and mutants denotes a difference of interest (not discrimination), which again could be due to any number of reasons.

We have now updated the analysis and interpretation (Fig 4E and lines 262-265). We show that indeed the mutant mice are able to significantly distinguish between novel and self scents, although the magnitude of the effect is significantly smaller than that of wild type. We have accordingly added additional interpretation and discussion that this could be due to reduced interest/motivation, discriminatory ability, or a combination of factors (lines 262-265).

Major 3: Is the investigation time for peanut butter and mouse urine significantly different from water? I don’t see any statistical tests for this comparison. This is another key point for the authors conclusions.

We have now added two statistical analyses: first, comparing wild-type to Stoml3 mutant investigation times, in which no significant differences are observed. Second, comparing within genotypes the investigation times of peanut butter or urine to water. Since some of the trends in these comparisons do not reach statistical significance, we have accordingly altered the corresponding interpretation of these experiments (Lines 232-236).

Minor 1: Could the authors please provide more details about the analysis of this data? Was the data analyzed by an observer blind to genotype etc? Did the authors measure nasal contact or just X distance from the source? Some of these details are included for the innate attraction test but are missing from the other behavioral assays. The number of animals should be included in the behavioral descriptions.

These details have now been added uniformly for each behavioral assay, with information provided in both the figure legend for Figure 4 and in each respective methods section. For details common to all assays (e.g. that the data was analyzed by an observer blind to genotype), these details are provided in lines 353-357.

Minor 2: The Y axis for Figure 4 should be investigation time, not sniffing. Mice are also sniffing when they are not near the stimulus. The error bars are not mentioned - are they stdev? For panels A,B,C, and E individual data points would be more impactful.

Figure 4 Y axes have now been updated, and Figure 4 legend now reports that error bars represent S.E.M. We have also overlaid individual data points for panels 4A, B, C, and E.

Minor 3: Given the widespread expression of stoml3, could the authors provide some behavioral evidence that these animals are relatively normal.

We have now added some additional observational evidence that the Stoml3 mutant animals are normal, both from our observations and from more targeted in-depth behavioral analysis in previous analyses (Lines 224-227).

Minor Concerns

Line 30 - FYI - more recent work has called into question the ability of humans to discriminate 1 trillion olfactory stimuli. Gerkin and Castro, 2015 eLife and others.

We have now edited the phrasing in the introduction to account for these points (lines 28-29).

Line 134 - Although I don’t deem this experiment necessary - I wonder whether the authors could provide more evidence for or against the minimal contribution of unc-1 and sto-4 for olfactory behavior by performing an analogous experiment to “smell the stick” with a non-olfactory stimulus (e.g., heat or light?). A similar reduction in locomotion would support that these genes are making a larger contribution to locomotor ability, while a similar locomotor response to wts could provide evidence for a role in olfactory behavior.

We have done some preliminary experiments very similar to those suggested, out of curiosity, using heat as a stimulus. Indeed, both unc-1 and sto-4 are poor at thermotaxis as well, suggesting an overall locomotory ability defect. However, we are still working to perfect this experimental paradigm in the lab.
