# Supplementary figures and images for: A Conserved Role for Stomatin Domain Genes in Olfactory Behavior
Source: eNeuro. 2023 Mar 21;10(3):ENEURO.0457-22.2023. doi: 10.1523/ENEURO.0457-22.2023 (PMC10035767; doi:10.1523/ENEURO.0457-22.2023)

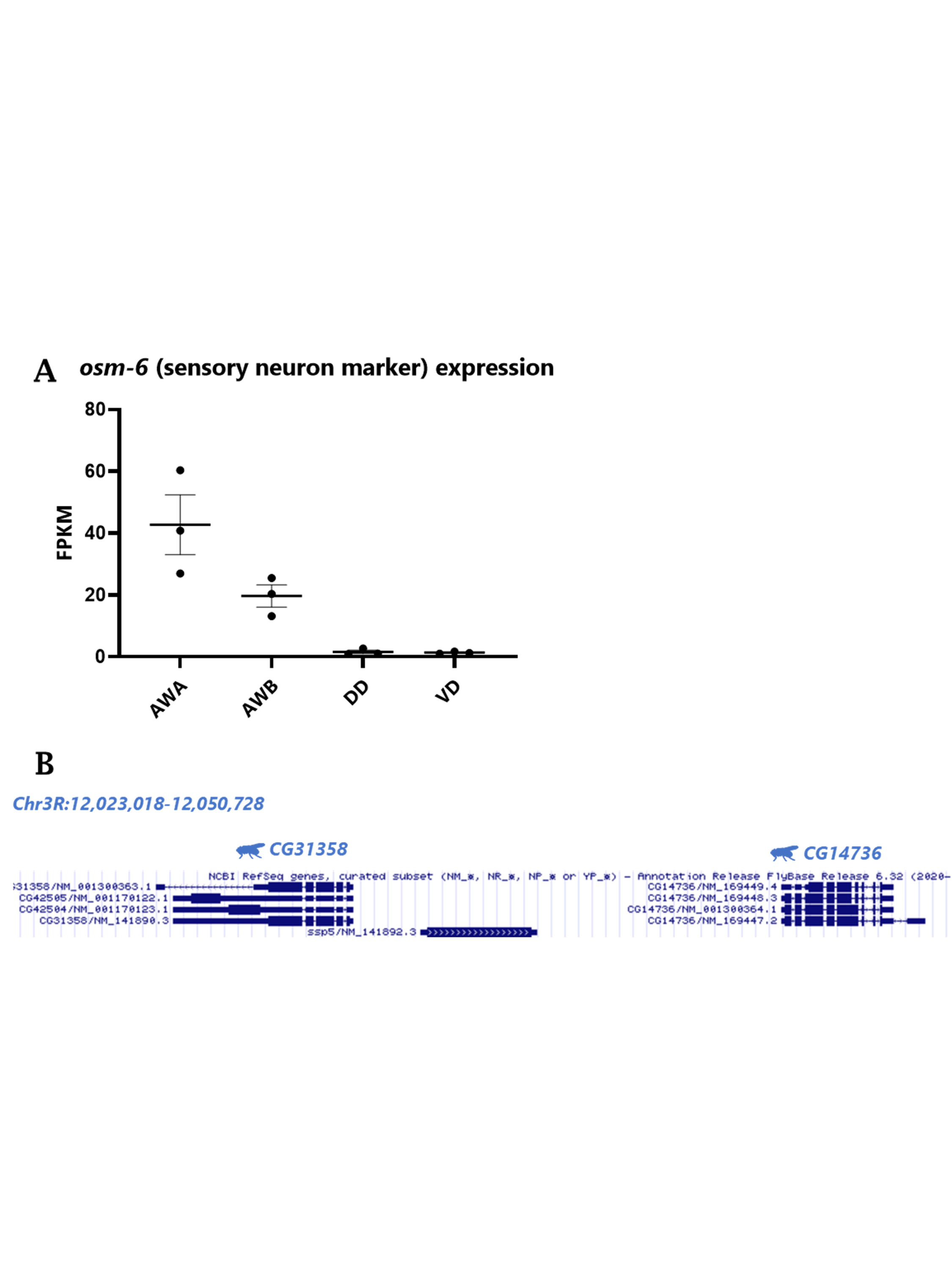

Supplement: Extended Data Figure 1-1 — Expression and location of Stomatin domain gene homologues. A, FPKMs for osm-6 as a positive control for a gene highly expressed in olfactory neurons (it serves as a sensory neuron marker gene) and lowly expressed in other neurons, including motor neurons. B, Additional cluster of Drosophila Podocin homologues on chromosome 3. Download Figure 1-1, TIF file. [file enu-eN-NWR-0457-22-s02.tif]

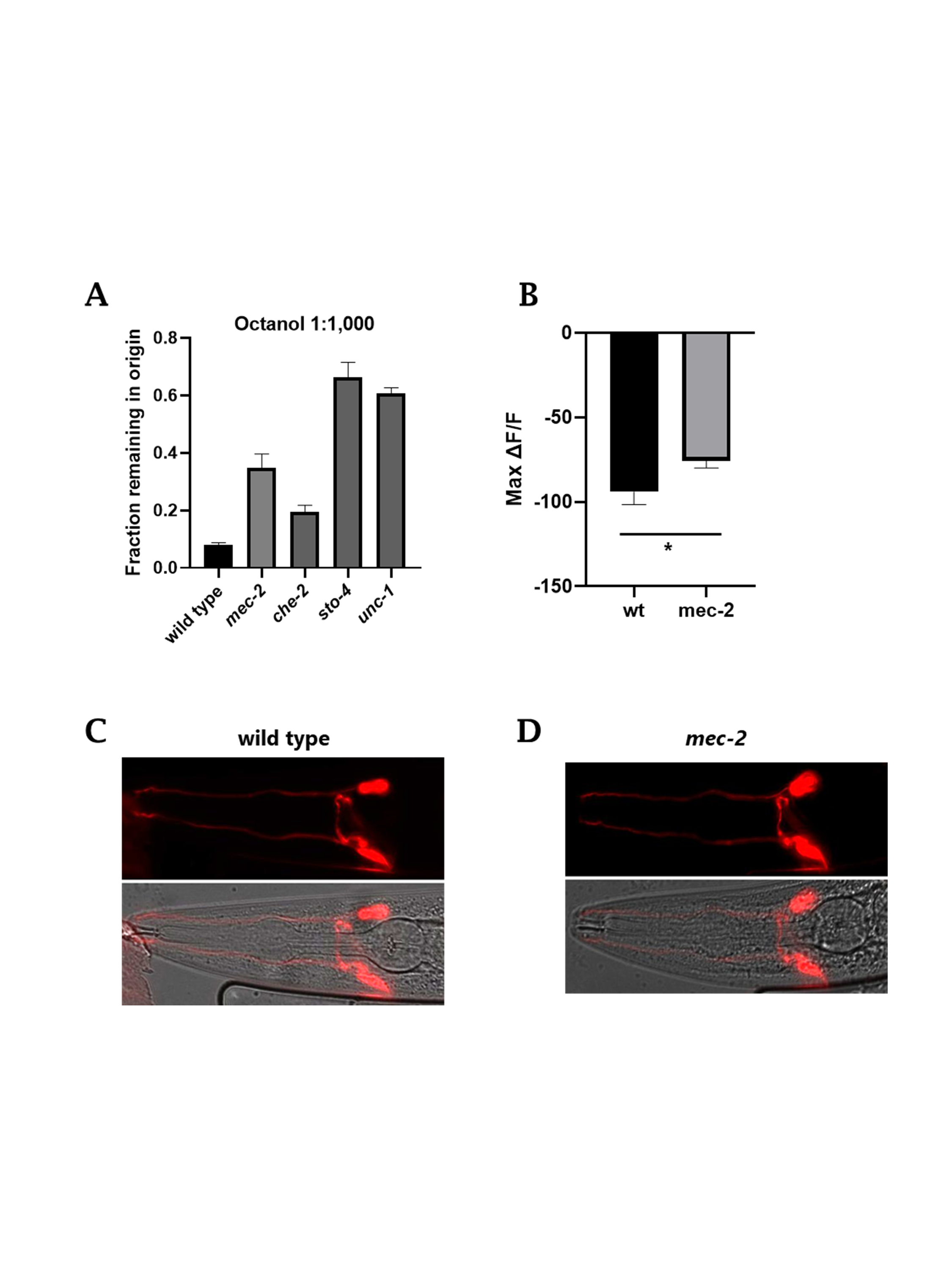

Supplement: Extended Data Figure 2-1 — Stomatin domain genes required for behavior in C. elegans. A, Fraction of worms that fail to leave the 1-cm diameter origin circle after 1 h. Note that the majority of sto-4 and unc-1 mutants fail to locomote away from origin, whereas both wild-type and chemotaxis defective mutants do not remain in origin. B, Maximal ΔF/F GCaMP signals during odor presentation of isoamyl alcohol (1:1000) as displayed in Figure 2G. Unpaired two-tail t test, p < 0.05. C, D, AWB and AWC neuronal cell bodies visualized by transgene oyIs44 (ord-1p::RFP), revealing no obvious morphological differences in cell body position, axons, or dendrites between wild-type (C) and mec-2 mutant worms (D). Download Figure 2-1, TIF file. [file enu-eN-NWR-0457-22-s03.tif]

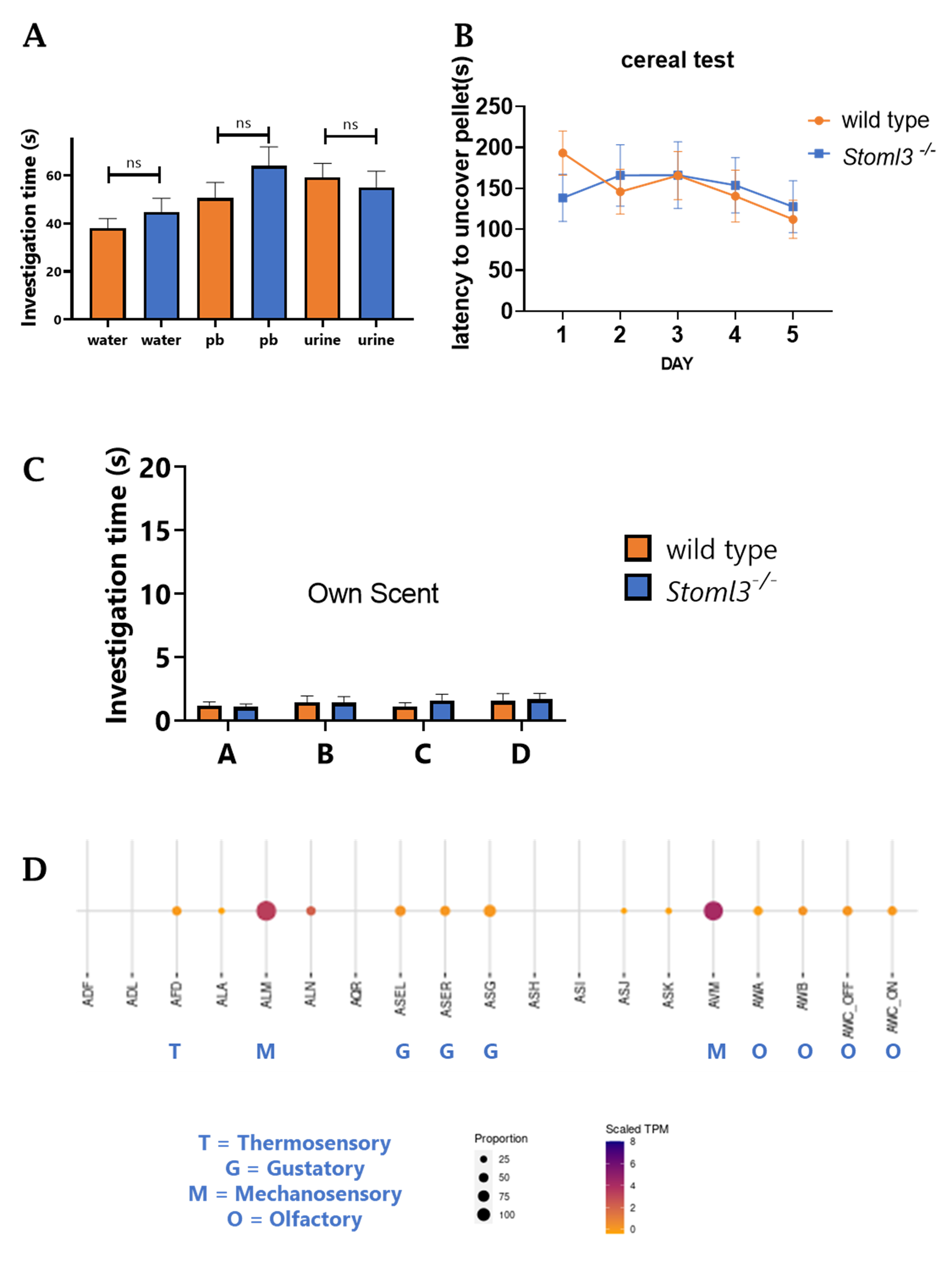

Supplement: Extended Data Figure 4-1 — Stoml3 behavior and mec-2 expression. A, Latency to uncover pellet in cereal test measured on a daily basis, showing no significant differences between dynamics of wild-type and Stoml3 KO mice. B, In trial 6 of the block test, where no novel odor is present, time spent exploring the blocks is minimal. C, Single-cell sequencing data from CenGEN consortium data on mec-2 expression in representative neuron populations. Size of dot represents proportion of single cells in which mec-2 was detected, and heatmap represents scaled TPM of the mec-2 gene. A few sensory cell types are highlighted. Download Figure 4-1, TIF file. [file enu-eN-NWR-0457-22-s04.tif]
